# Endometrial Receptivity Array (ERA) Test in a 32-Year-Old Female With Refractory Infertility: A Case Report

**DOI:** 10.7759/cureus.55703

**Published:** 2024-03-07

**Authors:** Sayaka Imoto, Miyako Funabiki, Yoshitaka Nakamura, Sagiri Taguchi

**Affiliations:** 1 IVF Center, Oak Clinic, Osaka, JPN

**Keywords:** ivf, live birth, refractory infertility, clinical efficacy, endometrial receptivity array (era) test

## Abstract

We report a successful case for the clinical efficacy of the endometrial receptivity array (ERA) test for a 32-year-old female patient with refractory infertility. After our careful treatment, the patient gave birth to a male baby (3390 g) in November 2021. To our knowledge, though the clinical efficacy of the ERA test is controversial until March 2024, we think that the age of the patients, the number of IVF cycles, and the racial differences may have an impact on the clinical efficacy of the ERA test.

## Introduction

There is a hypothesis that the endometrium has "window of implantation," a period of time when the endometrium is suitable for implantation [1,2].

According to this hypothesis [1,2], if the window of implantation and the time of implantation are misaligned, even if a good embryo is transferred to the uterus, pregnancy will not occur. Unfortunately, however, it is not possible to tell by ultrasound or internal examination whether the window of implantation is open or not.

The ERA (endometrial receptivity array) test is used to estimate the state of the endometrium, which cannot be determined by external observation [3]. To determine if the window of implantation is open, gene expression is examined by the ERA test.

Therefore, we examined the expression profiles of 238 genes expressed in the endometrium and examined the relationship between expression patterns and timing by the ERA test [3]. Our clinic conducted a randomized controlled trial (RCT) in collaboration with Prof. Simon's group to investigate the clinical efficacy of the ERA test (ERA-RCT) [3]. The results of the ERA-RCT showed that the ERA was effective [3].

Furthermore, the result of a meta-analysis, limited to RCTs, showed that the implantation rates, the clinical pregnancy rates, and the delivery rates were improved by the ERA test [4]. However, a recent RCT has failed to show any improvement in the above outcomes [5]. Therefore, the clinical efficacy of the ERA test may be limited, and further research is needed.

## Case presentation

The female patient was 32 years old at the time of her first visit to our clinic and had a menstrual cycle of 28 days and a BMI of 21.5. Though she had undergone timing methods and artificial insemination at another clinic for a year and a half, she could not conceive and was transferred to our clinic. She was diagnosed with functional infertility and began treatment with in vitro fertilization (IVF) at our clinic. As for the infertile patient, ovarian stimulation was performed with the following protocol at our clinic.

 As for the short gonadotropin-releasing hormone (GnRH)-agonist method, patients received a nasal spray of GnRH agonist (1800 µg /day, Fuji Pharma Co., Ltd, Toyama, Japan) from the first day of the cycle. In the protocol, follicle-stimulating hormone (FSH: Merck Biopharma Co., Ltd, Tokyo, Japan) or human menopausal gonadotropin (HMG: FERRING PHARMACEUTICALS, Tokyo, Japan) was administered daily from day 3. GnRH agonist was continued until human chorionic gonadotropin (hCG: Fuji Pharma Co., Ltd, Toyama, Japan) administration. The details were as follows: Suppression: GnRH agonist from Day 1 to GnRH agonist (buserelin acetate® three times a day two pushes total, once in each nose per dose (300㎍ x 3 times a day); Stimulation: Days 3 and 4 (two days): Forilmon P® 300 IU/day, Days 5-10 (six days): HMG 300IU/day, and Day 11 (one day): HMG 150IU/day.

The oocytes after retrieval by our stimulation protocol were subjected to conventional insemination or intra cytoplasmic sperm injection (ICSI). The retrieved oocytes in the present study were 23. On the following day, fertilization was confirmed by the presence of two pronuclei (2PN).

 As for embryo development and transfer, we improved as follows. We have judged the human blastocyst developments from the human embryos based on the Gardner and Schoolcraft scoring system [6]. The human blastocyst development was scored by the blastocoel stage (highest score is six), ICM grade (highest score is A), and trophectoderm (TE) grade (highest score is A) [6]. Therefore, human blastocysts with a score of 5BB [6] or higher were judged as good embryos at our clinic. In the first cycle of the present study at our clinic, five good blastocysts (human blastocysts with a score of 5BB [6] or higher) were transferred, but no pregnancy occurred. Furthermore, in the second cycle at our clinic, another good embryo (human blastocysts with a score of 5BB [6] or higher) was transferred, but no pregnancy was achieved. 

Therefore, we suspected that the window of implantation may be shifted and performed the ERA test to try to improve the clinical outcome of the patient, according to the approval of the institutional review board at Oak Clinic, Japan. As for the ERA Test methodology, the same method as in the study by Fujishima et al. was used in the present study [7]. 

**Figure 1:**
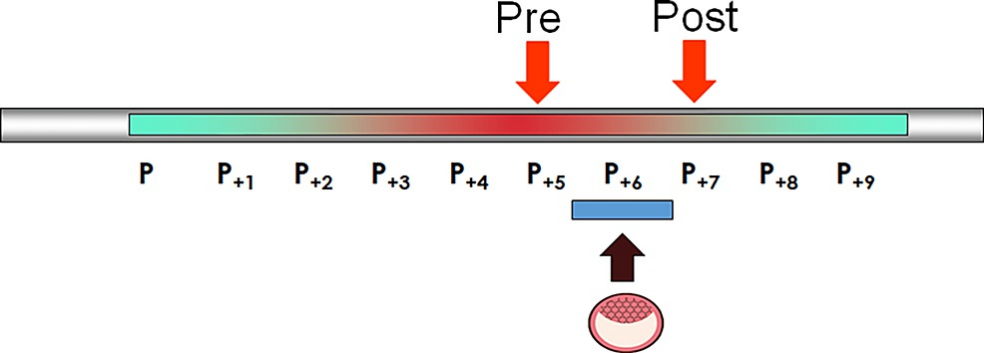
Results of the ERA test ERA: Endometrial receptivity array

 Figure 1 shows the result of the ERA test. The fifth day was PRE-RECEPTIVE and the seventh day was POST-RECEPTIVE (Figure 1). Therefore, we corrected the transfer date and transferred a good blastocyst (5BB) [6], which was considered optimal.

**Figure 2:**
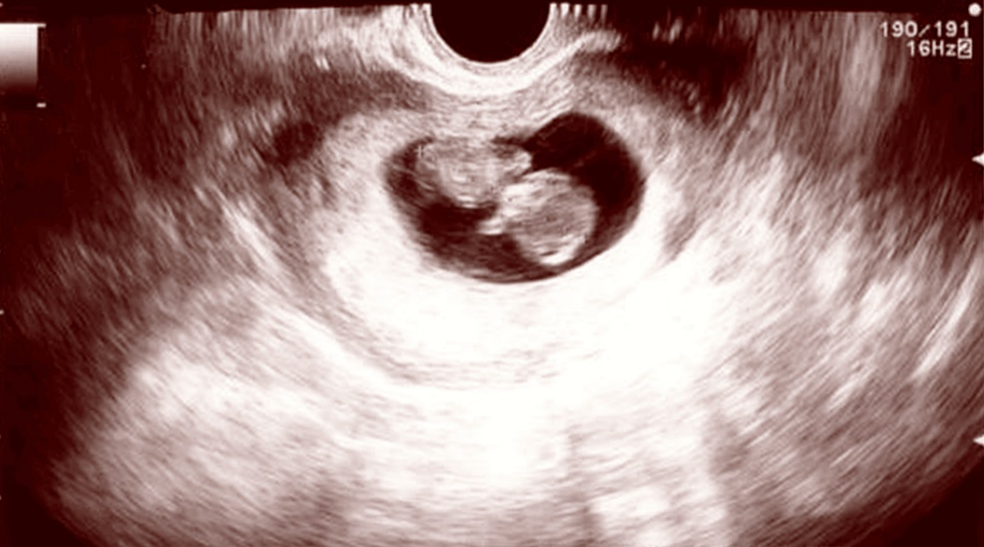
An ultrasound image of her pregnancy

As a result, pregnancy was achieved on the second transfer (Figure 2). Then, her hCG level at the time of pregnancy determination was 98.79 mIU/mL. Finally, a male baby (3390 g) was born in November 2021.

## Discussion

The clinical efficacy of the ERA test is controversial [3,4,5,7]. However, we could observe a live birth case for a female patient with refractory infertility by using the ERA test in the present case.

The patient selection criteria for the ERA-RCT in which we participated included an age range of less than 37 years [3] and the patient age in the present case was 32-year-old. Furthermore, we reported in our previous manuscript that the benefit of the ERA test was not always demonstrated in older patients (39 years old) [7].

The present case was not included in the ERA-RCT [3]. Furthermore, the present case was a relatively young patient in the age range included in the patient selection criteria of the ERA-RCT [3], and despite repeated transfers of good embryos (5BB) [6], pregnancy and delivery without the ERA test were not observed.

Based on the above, we think and investigate the age-dependent nature of the ERA test's clinical efficacy, including potential biological and physiological factors that may contribute to age-related differences in treatment outcomes. Currently, the ERA test's clinical efficacy may be associated with the age factor, the number of IVF cycles, and racial differences, when we consider our presentation study, a previous study [7], and past ERA-RCT data [3] in Japanese only as the data analysis approaches, although the data analysis is ongoing.

In addition, when considered in conjunction with the results of the meta-analysis for the ERA test, the clinical benefit of the ERA test may be limited, and there is an increasing need to closely examine "which patients receive the clinical benefit of the ERA test".

## Conclusions

In conclusion, we think that the age of the patients, the number of IVF cycles, and racial differences may have an impact on the clinical efficacy of the ERA test. Many manuscripts for the ERA test cases underscore the potential impact of these factors on the test's clinical efficacy and highlight the need for further research in this area. Therefore, we are investigating the associations among the age of the patients, the number of IVF cycles, the racial differences, and the ERA test by using clinical data at our clinic in Japan.
